# Colorimetric and Photobiological Properties of Light Transmitted Through Low-Vision Filters: Simulated Potential Impact on ipRGCs Responses Considering Crystalline Lens Aging

**DOI:** 10.3390/life15020261

**Published:** 2025-02-08

**Authors:** Ana Sanchez-Cano, Elvira Orduna-Hospital, Justiniano Aporta

**Affiliations:** 1 Department of Applied Physics, University of Zaragoza, 50009 Zaragoza, Spain; aporta@unizar.es; 2 Aragon Institute for Health Research (IIS Aragon), 50009 Zaragoza, Spain

**Keywords:** low-vision filters, aging crystalline lens, circadian rhythm

## Abstract

This study aims to investigate the potential impact of commercial low-vision filters on intrinsically photosensitive retinal ganglion cells (ipRGCs), which have significantly advanced our understanding of non-image-forming visual functions. A comprehensive analysis by modeling the potential responses of ipRGCs to commercially available low-vision filters was conducted, focusing on how the spectral properties of these filters could alter ipRGC function. Additionally, the influence of aging on the crystalline lens was considered. Colorimetric changes in the transmitted light by these filters were also analyzed, highlighting variations based on the manufacturer. The study uncovered the diverse responses of ipRGCs to fifty low-vision filters, shedding light on the potential modifications in ipRGC stimulation and visual function. Notably, the consideration of aging in the crystalline lens revealed significant alterations in ipRGC response. Furthermore, the analysis of colorimetric changes demonstrated substantial differences in the light transmitted by these filters, with variations dependent on the manufacturer. This research underscores the nuanced relationship between low-vision filters and ipRGCs, providing insights into their potential impact on visual function. The varying responses observed, coupled with the influence of aging on the crystalline lens, emphasize the complexity of this interaction. Additionally, the distinct colorimetric changes based on filter manufacturer suggest the need for tailored approaches in enhancing visual perception for individuals with visual impairments.

## 1. Introduction

The light and color quality transmitted through low-vision filters has not been extensively studied from a technical perspective [1]. Visual parameters such as visual acuity (VA) and contrast sensitivity have been noted as important subjective parameters to measure and quantify in people with low vision [2], along with their opinions and comfort regarding the use of low-vision filters; these variables have been parametrized by validated questionnaires [3,4,5]. Even if these filters have limited color fidelity, they have a noteworthy and undervalued ability to improve contrast and subjective comfort [3]. Selective filters contribute substantially to improving the quality of life of patients with low vision and maximizing their visual function. There is no standardized protocol for the prescription of filters in daily clinical practice according to their technical characteristics. Although a complete visual evaluation and preliminary questionnaire on the needs and difficulties of the patient in their daily life are carried out, it is also relevant to consider the lighting conditions and activity for which certain filters will be used [6].

About the impact of light on various ocular structures, it is important to note that short wavelengths (below 400 nm) have the potential to increase the risk of cataract formation [7,8]. Additionally, blue light, specifically within the range of 415 nm to 455 nm, has been identified as a potential cause of retinal damage [9,10]. Therefore, providing protection against blue light using blue-blocking sunglasses with yellow/amber lenses may be of significance for individuals who are prone to macular degeneration [11]. It has been reported that the visual system can be characterized as young only up to 40 years of age. Changes in the structures that make up the optical system of the human eye decrease the amount of light that reaches the retina; a classic symptom is a gradual, painless, progressive reduction in the quality of vision [12]. Hardening of the crystalline lens fibers and minimal changes in the ciliary muscles with age are the main causes of accommodation loss, in addition to changes in the thickness and transparency of the lens, which becomes yellowish with aging and thus develops age-related cataracts [13], and the pupil also becoming smaller [14]. This means that a significant portion of the changes in the lens caused by short wavelength exposure occur early in life, with substantial changes continuing throughout adulthood [15]. It is estimated that at 60 years of age, the retina only receives approximately one-third of the illuminance that a 20-year-old person’s retina does, requiring 3 to 10 times more light; the prevalence of age-related cataracts among people over 65 years of age in high-income countries is estimated at 30% [16].

Light plays a critical role in regulating human circadian rhythms and sleep. Ocular pathologies and blindness have been classically associated with circadian rhythm disturbances, particularly when the ability to perceive light is significantly impaired [17,18]. So, the impacts of light on health have been increasingly recognized and understood in recent years. Intrinsically photosensitive retinal ganglion cells (ipRGCs) and their photopigment melanopsin have been the subject of extensive study from various perspectives. While ipRGCs primarily contribute to non-image-forming visual functions, they also play an indirect role in modulating visual perception [18]. Their most well-established influence is on contrast sensitivity, enhancing the detection of subtle differences in light and dark areas. However, their contribution to color discrimination and spatial resolution remains less well understood and appears to be more limited. They participate in image-forming vision like other ganglion cells and respond to light through their endogenous photopigment melanopsin as well as rod/cone-driven synaptic inputs [19,20]. Additionally, ipRGCs also have a crucial role in non-image-forming visual functions, circadian photoentrainment, and are responsible for the pupillary light reflex; this reflex helps to regulate the amount of light entering the eye and ensures optimal visual function in varying lighting conditions [21,22,23]. Some studies suggest correlations between circadian rhythm disorders and retinal diseases such as glaucoma, diabetic retinopathy, or retinal ischemia, diseases in which circadian rhythm alterations have been experimentally linked to the impairment and loss of melanopsin-containing retinal ganglion cells in the retina, though the evidence for this remains limited [17].

Visual filters, through their ability to selectively manipulate the spectral properties of light, can influence ipRGC activation and potentially enhance or worsen visual function in individuals. Spitschan et al. [1] evaluated a wide selection of filters from a technical point of view, emphasizing spectral analysis to understand these physiological effects. Blue light filters, such as yellow-tinted lenses, should be worn at night, rather than during the day, because they can reduce the exposure of ipRGCs to excessive short wavelengths, helping to regulate circadian rhythm and reduce the risk of sleep disturbances [24]. They are recommended as low-vision filters because they can enhance contrast sensitivity by selectively increasing the transmission of specific wavelengths associated with high contrast perception, increasing the visibility of details that may otherwise be indistinguishable, or by modifying the spectrum of transmitted light and attenuating specific wavelengths to reduce the intensity of glare-inducing light [24].

From a technical perspective, several significant quantities are defined based on the spectral power distribution (SPD) of light transmitted by a given filter to quantify it. These include the melanopic equivalent daylight (D65) illuminance (mel-EDI) from the Commission International de l’Eclairage (CIE) S 026 [25,26,27], the equivalent melanopic lux (EML) from the WELL Building Standard v2 [28], and circadian stimulus (CS) [29,30]. The biological potential of light from 380 nm to 780 nm is based on the amount of light reaching the retinal plane, and the strength of the effect depends on the spectrum, intensity, duration, and timing of exposure and the spatial pattern of the light stimulus [31,32].

In addition, the colorimetric characteristics of the materials are also calculated from the SPD. The CIE General Color Rendering Index (CRI) [33] seems to be poor at describing the color rendering properties of daylight transmitted through materials. To complement this traditional method, the Illuminating Engineering Society (IES)’s Technical Memorandum 30 (ANSI/IES TM-30-20) [34] has been incorporated into different fields of both industry and research for evaluating colorimetric performance with transmitted light [35]. To calculate these quantities accurately, it is necessary to consider several factors; these include the SPD of the light source, the transmittance of the low-vision filter, and even the transmittance of an aging crystalline lens [36]. By considering these variables, it becomes possible to personalize and assess potential biological responses with greater accuracy, though individual responses may still vary due to intrinsic factors not fully accounted for by these calculations.

The main aim of this paper is to analyze the optical performance of a collection of fifty optical low-vision filters from different manufacturers using ANSI/TM-30-20 [34] and CIE 13.3 [33]. In addition, these filters under study were characterized with quantities from the CIE S 026 [25], UL 24480 [34] and WELL v2 [28] to evaluate their photobiological potential, particularly considering the variation in light transmission due to the aging of the crystalline lens and the potential development of age-related cataracts [37].

## 2. Materials and Methods

### 2.1. Filter Spectral Transmittance

A variety of 50 filters from AVS, Essilor, HOYA, ML, Prats, and Zeiss, commonly available for purchase, are typically employed as part of routine optometric assessments in daily clinics for low-vision rehabilitation. These manufacturers were selected as they represent some of the most widely prescribed brands for low-vision filters in Spain, based on our clinical experience and a review of common prescription practices in our region. Our objective was to obtain and measure all the available filters from these companies to ensure a comprehensive spectral and photobiological analysis. However, we acknowledge that some filters from other manufacturers may not have been included due to stock unavailability or logistical constraints related to shipments. They have been spectrally measured as part of this study to evaluate their colorimetric and the photobiological properties of the light transmitted through them. The experimental measurements were performed using a calibrated spectroradiometer (model StellarNet-Black Comet, StellarNet, Inc., Tampa, FL, USA with C20080502 calibration and NIST traceability. The uncertainty of measurement across the wavelength was ±0.5 nm and ±2.0% for transmittance). It was used for analyzing the filter transmittance from 380 nm to 780 nm in increments of 1 nm. The SPD of standard illuminant D65, 6500K correlated color temperature (CCT), was selected as a reference to calculate photopic parameters such as the filters’ transmittance *T* (%), Equation (1) [38], and to facilitate comparison among different filters. The selection of 50 filters analyzed is shown in Table 1.

(1) $$T\left(\%\right)=100\times \frac{\int_{\lambda =380}^{780}SPD\left(\lambda \right)\times \tau \left(\lambda \right)\times V\left(\lambda \right)\times d\lambda }{\int_{\lambda =380}^{780}SPD\left(\lambda \right)\times V\left(\lambda \right)\times d\lambda }$$

### 2.2. Colorimetric Properties

To compute colorimetric characteristics, the CRI was considered; the CRI measures color fidelity, which describes the average deviation between the test source and reference source for the Test Color Samples (TCSs), and R9 measures color fidelity for the 9th TCS (“saturated red”) of CIE 13.3 [33]. Additionally, the CIE chromaticity coordinates (x,y) of CIE 1931 and their chromaticity diagram were calculated and plotted.

To complete these parameters and to calculate the color rendering properties of the transmitted light, the ANSI/IES TM-30-20 Advanced Calculation Tool v 2.04, available for download at http://www.ies.org (accessed on 16 December 2024), was used to compute color rendering information according to IES TM-30-18 Annex D. Additionally, the paper by Houser et al. [35] served as a toolbox to upload the spectral transmittance of every filter. In the TM-30 document, the Fidelity Index (Rf), from 0 to 100, characterizes the mean similarity to a reference source for the 99 color evaluation samples (CESs). The Gamut Index (Rg), from 0 to 150, represents the average change in chroma for the 99 CESs. The Rcs,h1 value, from –95% to +85%, is a measure of the local chroma shift for the reddish CESs that plots within hue-angle bin 1 (out of 16). The Rf,h1 parameter, ranging from 0 to 100, is a measure of local color fidelity for the reddish CESs that is plotted within hue-angle bin 1 [34].

### 2.3. Photobiological Properties

**Figure 1:**
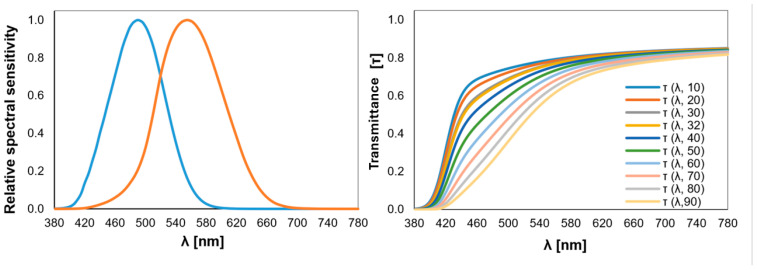
**Left**: The relative spectral sensitivities of melanopic (blue) and photopic (orange) light. **Right**: The spectral transmittances of the crystalline lens depending on age according to the NPR-CEN/TR 16791 document [37]. This figure shows the transmittance for each decade of life, with a 32-year-old subject as the standard. It can be observed that the older the individual is, the greater the absorption of lower wavelengths by the crystalline lens.

The circadian parameters of the light transmitted through the low-vision filters were calculated to evaluate their photobiological potential. These include mel-EDI under CIE S 026 E:2018 [25], EML from the WELL Building Standard v2 [28], and CS from UL 24480, each calculated following their respective methods [29]. It should be noted that mel-EDI is measured in lux, while EML is reported in “m-lux”, a term used by the WELL Building Standard but not a standardized unit according to the International System of Units (SI). CS is a measure of the amount of human nocturnal melatonin suppression from a light stimulus with a specified spectrum and intensity. The duration of exposure is assumed to be 1 h. CS has a range of 0.0 (no nocturnal melatonin suppression) to 0.7 (70% nocturnal melatonin suppression). With the consideration described above, a fixed photopic illuminance of 250 lx at the corneal plane was selected to compute the values for mel-EDI, EML, and CS. After that, mel-EDI and EML were estimated at the retinal plane considering the potential transmittance of an aging crystalline lens (Figure 1). To do this, the NPR-CEN/TR 16791 document [37] was used to consider the aging of the crystalline lens to compute variations from standard 10- to 80-year-old observers.

### 2.4. Risk of Bias

While this study focuses on objective measurements, potential biases exist. The selection of filter brands was limited to the most common in Spain (AVS, Essilor, HOYA, ML, Prats, and Zeiss) due to availability, logistics, and funding, excluding others (Cocoons, Chadwick Optical, etc.). This may limit the generalizability of results. A single calibrated spectroradiometer was used; multiple measurements and consistent protocols mitigated potential bias. Computational methods, while based on standards, may introduce bias. Widely accepted tools and methods were used, and parameters were stated for transparency.

## 3. Results

**Figure 2:**
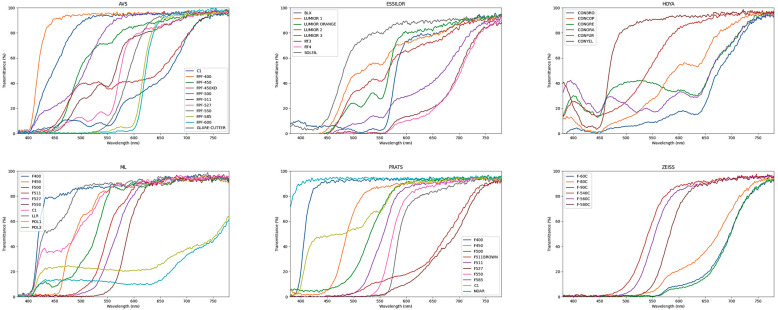
The spectral transmittance from 380 nm to 780 nm for the different low-vision filters analyzed. In order from top to bottom, the graphs correspond to AVS, ESSILOR, HOYA, ML, PRATS, and ZEISS.

The spectral transmittances of the evaluated filters, detailed in Table 2 and Figure 2, are rather different, even when the filters share a name. Diversity is manifested in the form of the filters’ spectral transmittance function and the cutoff wavelength, and they exhibit significant variation in the wavelengths of light that they transmit.

**Figure 3:**
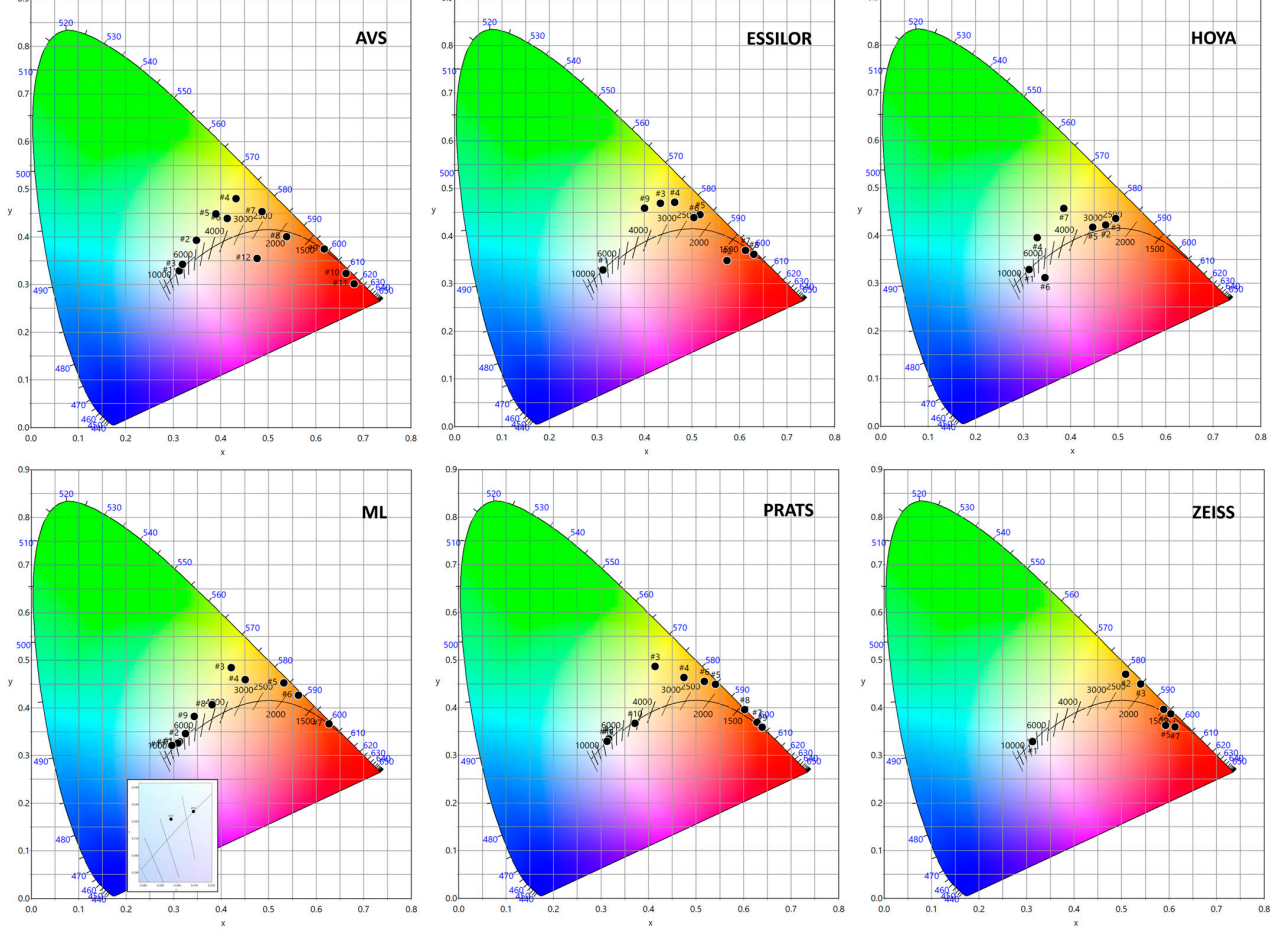
A chromatic diagram representing the location of D65 and the low-vision filters evaluated. The numbers “#n” are the different filters enumerated in Table 2.

All filters exhibit a shift in chromaticity from the D65 white point toward the upper spectral locus, which is represented by the boundary of the chromaticity diagram, as presented in Figure 3 and Table 2. As a consequence, the color rendering characteristics of these filters computed by both CIE 13.3 [33] and ANSI/TM-30-20 [34] considerations are greatly modified compared to the standard illuminant D65 (CRI = 100, Rf = 100, Rg = 100, Rf,h1 = 100, and Rcs,h1 (%) = 0). As Table 2 shows, shifts in color toward the spectral locus result in a diminished color gamut. In some cases, as shown in Table 2, these colorimetric properties cannot be computed following the standard rules due to the low spectral transmittance shown by some filters. The same behavior can be observed when analyzing CCT, as this parameter becomes impossible to calculate due to this characteristic.

An analysis of these low-vision filters under melanopic conditions, compared to the standard illuminant D65, is presented in Table 3. These results are computed for the eye of a 32-year-old observer, considered the standard eye, and compared using three globally recognized metrics. A photopic illuminance of 250 lx at the corneal level is equivalent to 250 mel-EDI, 275 EML, and 36.3% CS when D65 is used as the standard illuminant. Wearing some low-vision filters has a profound impact on the levels of melanopic light, which is contingent on their spectral transmittance. Even though the level of photopic light, after the low-vision filter, reaching the corneal plane remains at 250 lx, the calculated melanopic values reveal that this reduction can reach up to 95% for filters with very long cutoff wavelengths or lower transmittances, as summarized in Table 3.

**Figure 4:**
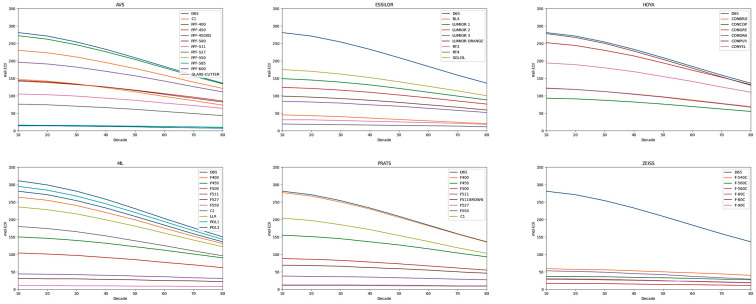
Melanopic lighting (mel-EDI) values as a function of age at the retinal plane after light passes through the filters, analyzed by filters, and compared to the values for the standard illuminant D65; from a 10-year-old observer to an 80-year-old observer, this is a representation of each decade of life, indicating in increments of 10 years.

When the aging of the crystalline lens is considered in this analysis, significant modifications in the level of melanopic light reaching the retinal plane are observed. To simplify the analysis, only the mel-EDI levels are presented, illustrating the influence of the transmittance of the lens as a function of age. When EML is needed, it can be easily calculated with the expression EML (m-lux) = 1.104 × mel-EDI [40]. The substantially reduced levels of melanopic light reaching the retina in older individuals should be taken into consideration, especially when recommending the use of filters for individuals with specific retinal pathologies. These findings highlight the importance of addressing decreased melanopic light sensitivity in older populations and the potential benefits of utilizing filters to compensate for these deficiencies (Figure 4).

## 4. Discussion

The efficacy of filters remains a subject of debate; certain studies suggest subjective visual enhancements or, in some instances, declines in visual function measures such as VA, contrast sensitivity, color vision, and relief of eye strain symptoms [24,41,42]. Both VA and contrast sensitivity have been subjectively studied in patients with low vision who used colored filters to improve their performance in daily tasks; however, these filters have not been studied objectively in depth [2,3]. In clinical practice, filters are prescribed to individuals with various retinal disorders, despite the absence of a standardized guideline on the selection and prescription of them. The impact of filters and lighting on the ability to perceive contrast depends on the reason for the decline in contrast sensitivity. It is necessary to consider the root cause of a patient’s visual impairment to provide tailored and individualized care that optimizes the patient’s well-being [24,43]. Relatedly, Bailie et al. [3] found that colored filters could reduce glare discomfort without affecting VA or contrast sensitivity in patients with age-related macular degeneration, and caused a small reduction in objective color vision not noticed subjectively by the patients. Domínguez-Vicent et al. studied the use of selective absorption filters in photopic glare and non-glare conditions, as well as mesopic glare conditions, and the contrast sensitivity measured with the filters did not show a significant difference from the measurement taken without filters in young healthy adults [44].

This novel study provides clinically relevant information by evaluating the potential optical and photobiological properties of low-vision filters, with a specific focus on the aging crystalline lens, in order to optimize the selection of a low-vision filter and form a more personalized approach for the visual rehabilitation of a person according to their pathology and age. Furthermore, the study is not limited to blue-blocking filters but covers a variety of filters with different spectral transmission properties, addressing the diverse visual needs of people with low vision. Although filters with the same name may appear to be the same color, they can have different spectral properties, as the results show, making them cases of metamerism. This is significant because non-image-forming effects can be adjusted by changing the spectrum while preserving acceptable visual appearance and maintaining consistent visual behavior tailored to the specific needs of each individual [45].

Variability in the results has been described by other authors who examined different types of colored tinted lenses and diverse causes of low vision [46,47]. Rosenblum et al. [48] found subjective improvement in VA, contrast sensitivity, and glare sensitivity in a group of patients with low vision, and there were also reductions in photophobia, eyestrain and eye discomfort, showing that any form of pathology needs its own specific spectral filter, or even additional neutral filters meant to be worn outdoors on sunny days. Tavazzi et al. [49] also studied the changes that patients presented in contrast sensitivity when they used filters; in this case, they studied two blue light filters that differ only in the presence of a band at 450 ± 20 nm in the transmittance spectrum. Patients had an improvement or worsening in contrast sensitivity depending on the contrast sensitivity they already had and the filter they used. Domínguez-Vicent et al. also stated that in mesopic settings without glare, the filters resulted in a reduction in contrast sensitivity [44].

As outlined, the discrepancies observed in previous studies regarding the effects of filters on visual functions can be attributed to several factors, including variations in the types of filters used, their overall transmittance, the specific tests employed to measure visual functions, and the levels of luminance and glare. In healthy subjects, there has been a demonstrated systematic reduction in photopic contrast sensitivity, which has been correlated with the transmittance of the filter. However, these tests often have a limited range of contrast levels, which means that they may not be sensitive enough to detect subtle changes; they also depend on the luminance level, as there is a notable dissociation between photopic and mesopic contrast sensitivity even in individuals with normal vision.

Several authors have studied the subjective effect that colored filters can have; in contrast to neutral density filters, colored filters differentially absorb different wavelengths across the visible spectrum, which usually causes color vision distortion. A similar deduction can be made from our results; the color rendering of transmitted light through the analyzed low-vision filters has been difficult to accurately analyze, since TM-30-20 was developed to characterize lights with SPDs near the Planckian locus. The transmitted light that was evaluated fell outside the bins specified in the ANSI C78.377-2017 documents [39], specifically in chromaticity, and in this case, the results should be interpreted with caution. These bins can appear slightly colored, and chromatic adaptation may not be accurately modeled since the evaluated filter and the reference illuminant have different chromaticities despite comparable Rf, Rg, and CCT values. Most of the analyzed filters have highly saturated SPDs (marked with * in Table 1), in concordance with the analysis performed by Spitschan et al. [1]; this property distorts some ranges of color and the chromatic adaptation needed to carry out certain activities. These filters, that selectively enhance or suppress certain color ranges, can modify color contrast and color recognition abilities and have a strong influence on daily life.

In pathologies such as albinism, photophobia is alleviated or treated by wearing unspecific tinted spectacles or filters, but there is little evidence as to which type of filter should be used or what effects different filters have [50]. There have been studies where most individuals showed a preference for neutral filters in shades of gray or brown, while only a small number favored filters with more noticeable colors such as orange. Colored filters can be deemed advantageous in ocular conditions characterized by cone dysfunction, as the dyschromatopsia induced by these filters may be less perceptible to the affected individual. However, individuals with albinism and normal color vision may find the dyschromatopsia to be disruptive and bothersome. The selection of filters differed even for indoor and outdoor glasses [51]. For instance, neutral density filters maintain a consistent reduction in light intensity across the entire visible spectrum. Alternatively, specific portions of the visible spectrum can be diminished by utilizing colored filters such as a yellow filter, which absorbs light more intensely in the shorter wavelength range. As the wavelength of light decreases, light scattering becomes more prominent. This phenomenon elucidates why colored filters might be employed to augment contrast vision, a valuable aspect in numerous low-vision rehabilitation scenarios. Nonetheless, the utilization of colored filters can potentially induce dyschromatopsia.

In the reviewed literature, the photobiological analyses of this type of filter are weak; from our results, low-vision filters could seriously disrupt circadian rhythms in people wearing them because they provide very low levels of light, independent of the metric used (as outlined in Table 2), compromising normal behavior and limiting people’s well-being. In practical observations, it has already been noted that individuals with congenital achromatopsia tend to exhibit a tendency toward a late chronotype. These individuals often experience pronounced light avoidance and photophobia when exposed to daylight levels of illumination. As a means of alleviating visual discomfort, they frequently utilize various spectral filters. In some cases, a combination of dual filters can be the solution to the dispute over the use of different filters depending on the lighting. Dual-filter systems are based on the combination of two filters: one based on low-light conditions and another as a supplementary adjustable filter designed to prevent glare [52]. This fact supports the justification of a personalized analysis of each condition. Since retinal irradiance is modulated by the ocular anterior pole of each person, to determine effective light exposure in people, pupil size, corneal irradiance, spectral filtering by filters, and inner media transmittance modulated by the aging lens should be considered as a whole.

Spitschan et al. [1] found that there is a correlation between the attenuation of melanopsin activation and spectral filters with reduced light transmittance at short wavelengths, in accordance with our results. Personalized custom-made glasses with the ability to minimize the light stimulation of ipRGCs have been proposed as a potential tool for mitigating migraine attacks by controlling the light environment surrounding people at night [53]; certainly, our results hint at the possibility of leveraging this control mechanism to explore strategies for optimizing the well-being of individuals with low vision.

Low-filter analysis has not been extensively discussed in the literature; meanwhile, it has been reported that color tests reveal minor worsening of blue/yellow color vision and worse color discrimination after wearing blue-blocking filters, and the substantial variability observed among different individuals suggests that the acceptance of these filters varies among people [54,55]. The long-term use of these filters seems to induce some degree of adaptation to the modified lighting conditions, although the effects are modest, and the variability is significant [56]. Additionally, different behaviors have been reported in commercial blue-blocking lenses depending on the spectral transmittance of the material, resulting in different grades of effects on melatonin suppression or even on blue color perception [57], while other studies based on orange-tinted blue-blocking glasses or custom-made filters support this melatonin suppression, trying to minimize changes in color perception at the same time [58,59,60]. To address filter limitations, other alternatives have been proposed; for this purpose, the spectral composition of a composite system was customized to meet the specific requirements of individual users by fine-tuning the contributions of primary light sources [61].

These results emphasize the variability in how different filters can affect non-image-forming functions, which are crucial for overall patient comfort and for tailoring personalized care. Previous clinical research has underscored the importance of understanding filter characteristics and their impact on visual performance, beyond merely improving visual function. Consequently, the clinical significance of our study lies in guiding the selection and prescription of filters tailored to individual needs, particularly for those with low vision or retinal disorders, ultimately enhancing quality of life by reducing glare and improving visual comfort. To compensate for age-related effects on filter performance, clinicians can customize selections based on patient profiles, including age and visual pathology, thereby optimizing filter effectiveness, and supporting visual rehabilitation in people.

This study has some limitations that should be acknowledged. While we aimed to provide a comprehensive analysis of low-vision filters, our focus was on the brands most commonly prescribed in Spain (AVS, Essilor, HOYA, ML, Prats, and Zeiss), based on our clinical experience and a review of common prescription practices in our region. Although we intended to include all commercially available filters from these manufacturers, we encountered several challenges during the data collection phase. These included limitations in stock availability, logistical constraints related to international shipments, and funding limitations, among other unforeseen issues. As a result, we were unable to acquire and analyze filters from all manufacturers globally, including well-known brands such as Indo, Cocoons, Chadwick Optical, Corning, and NoIR Medical Technologies, among others, despite our efforts. Future research could expand upon this work by including a broader range of filter brands and manufacturers to provide an even more comprehensive understanding of the spectral and photobiological properties of low-vision filters.

## 5. Conclusions

In conclusion, the evaluation of the interaction between ipRGCs and low-vision filters offers a novel approach to analyze the aids that individuals with visual impairments use. By selectively manipulating the spectral characteristics of light, low-vision filters could optimize ipRGC activation, thereby improving contrast sensitivity, reducing glare, and enhancing color discrimination and visual comfort. For example, improved contrast sensitivity could translate to better object recognition and navigation in low-light conditions, while reduced glare could enhance visual comfort and reduce eye strain during daily activities. This optimization does not imply enhancing blue light exposure specifically, but rather adjusting the spectral composition of light based on individual visual needs. The customization of filters based on individual needs and preferences seems to play a crucial role in optimizing their effectiveness and constitutes the main limitation to widespread implementation. Future investigations and technological innovations aimed at advancing visual rehabilitation and elevating quality of life for individuals with visual impairments are needed. Although the use of low-vision filters to enhance ipRGC-mediated visual perception appears promising, it is essential to explore new challenges for future development in this optometric specialty. These include optimizing filter design for specific visual conditions; conducting long-term studies to assess the efficacy and durability of low-vision filters; and exploring the potential synergies between low-vision filters, opacification, aging of the crystalline lens, and the mitigation of ocular pathologies. Perhaps further research should focus on developing standardized protocols for filter manufacturing and customization, optimizing filter designs to address a wider range of visual impairments, or exploring novel materials and technologies. Empirical investigations, especially clinical studies, are essential to validate these assertions, examining subjective perceptions and rigorously assessing light levels alongside their spectral attributes across diverse exposure durations. Further experimental research is warranted to comprehend how light exposure duration, spectral distribution, and the use of low-vision filters interact, necessitating a comprehensive exploration. Ultimately, our findings suggest that low-vision filters have the potential to significantly improve the quality of life for individuals with visual impairments by enhancing their visual function and comfort.

## Figures and Tables

**Table 1:** Selection of fifty low-vision filters analyzed by manufacturer.

Manufacturer	Commercial Name	Low-Vision Filters	
AVS	AVS Baja Visión (Madrid, Spain)	C1	FPF-527
		FPF-400	FPF-550
		FPF-450	FPF-585
		FPF-450XD	FPF-600
		FPF-500	GLARE-CUTTER
		FPF-511	
ESSILOR	Essilor International (Paris, France)	BLX	LUMIOR 3
		LUMIOR 1	RT3
		LUMIOR ORANGE	RT4
		LUMIOR 2	SOLEIL
HOYA	Hoya Corporation (Tokyo, Japan)	CONBRO	CONORA
		CONCOP	CONPUR
		CONGRE	CONYEL
ML	ML Optics (Madrid, Spain)	F400	F550
		F450	C1
		F500	LLR
		F511	POL1
		F527	POL3
PRATS	Grupo Prats (Barcelona, Spain)	F400	F527
		F450	F550
		F500	F585
		F511BROWN	C1
		F511	NOAR
ZEISS	Carl Zeiss (Jena, Germany)	F-60C	F-540C
		F-80C	F-560C
		F-90C	F-580C

**Table 2:** The photometric and colorimetric parameters (transmittance and color) for standard illuminant D65 and the low-vision filters analyzed. * represents analysis from ANSI C78.377-2017 [39] and # indicates that the results obtained with the TM-30 standard [34] should be considered with caution. + indicates that a filter was not evaluated since it is a standard filter (AR means antireflection coating).

			CCT (K)	CIE 13.3 CRI	x	y	Rf	Rg	Rf,h1	Rcs,h1 (%)	T (%)	
	#1	D65	6498	100	0.3128	0.3291	100	100	100	0	---	
D65 and AVS FILTERS	#2	C1	5006	91	0.3486	0.3928	90	92	91	−4	92.39	
	#3	FPF-400	6085	98	0.3195	0.3423	96	98	97	−1	95.02	*
	#4	FPF-450	3607	78	0.4318	0.4801	69	72	80	−6	71.88	*
	#5	FPF-450XD	4203	84	0.3898	0.4485	76	81	87	−1	39.01	*
	#6	FPF-500	3674	85	0.4139	0.4390	88	90	87	−7	79.09	*
	#7	FPF-511	2664	86	0.4864	0.4530	74	75	81	−6	49.46	*
	#8	FPF-527	1830	77	0.5388	0.4005	82	92	72	−13	35.93	*
	#9	FPF-550	1262	63	0.6179	0.3748	64	83	54	−23	24.03	*
	#10	FPF-585	---	---	0.6634	0.3232	7	82	0	40	9.44	#
	#11	FPF-600	---	---	0.6811	0.3015	5	89	0	41	7.12	#
	#12	GLARE-CUTTER	2060	78	0.4765	0.3545	84	106	88	−4	15.59	*
D65 and ESSILOR FILTERS	#2	BLX	1346	50	0.5733	0.3486	30	108	12	−32	25.47	*
	#3	LUMIOR 1	3513	81	0.4335	0.4685	69	73	80	−5	59.04	*
	#4	LUMIOR 2	3078	81	0.4636	0.4710	63	66	76	−5	48.31	*
	#5	LUMIOR 3	2286	88	0.5169	0.4450	68	68	82	−4	18.28	
	#6	LUMIOR ORANGE	2378	84	0.5036	0.4388	71	74	75	−9	45.98	
	#7	RT3	1262	60	0.6126	0.3697	57	93	38	−24	5.13	*
	#8	RT4	1154	58	0.6302	0.3616	57	91	47	−25	3.82	*
	#9	SOLEIL	4047	81	0.4001	0.4585	75	79	83	−6	79.54	*
D65 and HOYA FILTERS	#2	CONBRO	2603	97	0.4740	0.4227	91	94	94	1	10.84	
	#3	CONCOP	2452	87	0.4948	0.4359	88	87	86	−6	32.30	
	#4	CONGRE	5612	88	0.3294	0.3959	89	90	93	−2	37.73	*
	#5	CONORA	2955	88	0.4464	0.4178	91	96	89	−6	58.06	
	#6	CONPUR	4760	79	0.3463	0.3119	88	106	84	7	23.27	*
	#7	CONYEL	4325	78	0.3858	0.4570	72	76	81	−6	88.56	*
D65 and ML FILTERS	#2	F400	5837	98	0.3248	0.3456	97	99	98	0	88.10	
	#3	F450	3805	74	0.4211	0.4843	65	68	76	−7	80.39	*
	#4	F500	3187	78	0.4506	0.4591	84	86	82	−9	69.30	*
	#5	F511	2199	69	0.5317	0.4527	64	61	72	−12	50.54	*
	#6	F527	1812	67	0.5624	0.4265	65	61	66	−15	40.58	*
	#7	F550	1188	54	0.6269	0.3663	54	85	38	−31	23.51	*
	#8	C1	4181	89	0.3811	0.4069	92	94	91	−5	79.63	*
	#9	LLR	5151	93	0.3432	0.3820	93	95	93	−3	88.50	*
	#10	POL1	6715	95	0.3094	0.3259	94	101	92	4	21.81	
	#11	POL3	7574	96	0.2962	0.3214	93	100	90	6	11.61	*
D65 and PRATS FILTERS	#2	F400	6315	99	0.3156	0.3341	98	99	99	0	93.06	
	#3	F450	3945	74	0.4135	0.4868	68	70	78	−7	83.00	*
	#4	F500	2880	78	0.4746	0.4632	81	82	82	−9	64.84	*
	#5	F511	2103	66	0.5406	0.4493	53	49	66	−14	48.70	*
	#6	F511BROWN	2347	85	0.5174	0.4554	68	69	88	0	12.78	
	#7	F527	1194	80	0.6281	0.3689	75	61	72	−12	5.04	*
	#8	F550	1425	49	0.6025	0.3954	39	26	37	−26	31.23	*
	#9	F585	1106	43	0.6394	0.3586						#
	#10	C1	4196	96	0.3711	0.3668	96	99	96	−3	73.34	
	#11	NOAR	---	---	---	---	---	---	---	---	---	+
D65 and ZEISS FILTERS	#2	F-540C	2530	70	0.5086	0.4704	61	59	74	−11	58.06	*
	#3	F-560C	2114	66	0.5397	0.4500	55	50	67	−13	48.10	*
	#4	F-580C	1380	59	0.6035	0.3866	62	80	56	−22	28.03	*
	#5	F-60C	1323	76	0.5925	0.3631	62	123	47	−12	3.88	*
	#6	F-80C	1504	73	0.5883	0.3967	79	87	75	−12	10.03	*
	#7	F-90C	1215	74	0.6128	0.3592	52	136	36	−16	2.99	*

Abbreviations: CCT, correlated color temperature; CIE, Commission International de l’Eclairage; CRI, Color Rendering Index; Rf, Fidelity Index; Rg, Gamut Index; T, transmittance.

**Table 3:** The changes in circadian lighting (mel-EDI) for D65 reaching the retinal plane depending on the low-vision filter and the age of the subjects, considering 250 photopic lux at the corneal plane (wearing each filter). Calculations without age considerations correspond to a standard observer age of 32 years, as described in the current normative standards.

	FILTERS		mel-EDI									EML	CS (%)
	Years		10	20	30	40	50	60	70	80	32	32	32
	#1	D65	281	271	254	233	209	184	159	136	250	276	36.3
D65 and AVS	#2	C1	230	223	211	195	178	159	139	121	208	230	24.7
	#3	FPF-400	272	262	246	226	204	180	156	134	243	268	33.9
	#4	FPF-450	142	138	132	125	116	106	96	85	131	145	28.4
	#5	FPF-450XD	196	191	182	170	157	142	126	111	180	198	34.9
	#6	FPF-500	144	140	133	125	115	104	93	82	132	145	29.2
	#7	FPF-511	105	103	99	93	87	79	72	64	98	108	23.1
	#8	FPF-527	76	74	70	66	61	55	49	43	69	77	18.2
	#9	FPF-550	14	14	13	13	12	11	11	10	13	14	3.7
	#10	FPF-585	16	15	15	14	13	12	11	9	15	16	4.2
	#11	FPF-600	14	14	13	12	11	9	8	7	13	14	3.9
	#12	GLARE-CUTTER	147	142	134	123	111	98	86	73	132	146	30.0
D65 and ESSILOR	#2	BLX	45	43	40	36	32	28	24	20	39	44	12.4
	#3	LUMIOR 1	149	145	139	131	121	110	99	88	137	151	29.4
	#4	LUMIOR 2	124	121	116	110	102	94	85	76	115	127	25.9
	#5	LUMIOR 3	84	82	79	74	70	64	58	52	78	86	19.3
	#6	LUMIOR ORANGE	99	96	92	87	81	74	67	59	91	101	22.2
	#7	RT3	31	31	29	27	25	23	21	18	29	32	8.4
	#8	RT4	19	18	17	16	15	14	13	11	17	19	5.0
	#9	SOLEIL	175	170	162	152	140	127	114	100	160	177	32.6
D65 and HOYA	#2	CONBRO	121	118	112	105	97	88	78	69	111	123	25.9
	#3	CONCOP	93	91	87	82	76	69	62	55	86	95	21.3
	#4	CONGRE	252	244	230	214	194	173	153	132	227	251	27.3
	#5	CONORA	122	118	112	104	96	86	77	67	111	122	26.2
	#6	CONPUR	278	267	250	228	204	179	154	130	246	271	37.2
	#7	CONYEL	194	189	180	169	155	141	125	110	178	197	34.7
D65 and ML	#2	F400	264	255	240	220	199	175	152	131	236	261	33.0
	#3	F450	150	146	140	132	122	112	101	90	138	153	29.5
	#4	F500	104	101	97	91	85	77	70	62	96	106	23.1
	#5	F511	44	43	42	40	38	36	33	31	42	46	11.0
	#6	F527	31	30	30	28	27	25	24	22	29	32	7.9
	#7	F550	11	11	10	10	9	9	8	8	10	11	2.9
	#8	C1	180	174	165	153	139	125	110	96	163	179	17.2
	#9	LLR	236	228	216	199	181	161	141	122	213	235	26.6
	#10	POL1	295	284	267	245	220	194	168	143	263	290	36.9
	#11	POL3	311	299	281	258	231	203	176	150	276	305	38.4
D65 and PRATS	#2	F400	277	267	250	230	206	182	158	135	247	272	35.3
	#3	F450	155	151	145	136	127	116	104	93	143	158	30.1
	#4	F500	88	86	83	78	73	67	61	55	82	90	20.2
	#5	F511	38	37	36	35	33	31	29	27	36	40	9.5
	#6	F511BROWN	69	68	66	62	59	55	50	46	65	72	16.4
	#7	F527	13	13	13	12	12	11	10	10	13	14	3.4
	#8	F550	11	11	11	11	10	10	9	9	11	12	3.0
	#9	F585	---	---	---	---	---	---	---	---	---	---	---
	#10	C1	204	197	185	171	154	137	119	103	183	202	25.2
D65 and ZEISS	#2	F-540C	59	57	56	53	50	47	44	40	55	61	14.2
	#3	F-560C	37	37	36	34	33	31	29	27	35	39	9.4
	#4	F-580C	17	16	16	15	14	13	12	11	16	17	4.4
	#5	F-60C	53	51	49	45	42	37	33	29	48	53	13.6
	#6	F-80C	29	28	27	26	24	23	21	19	27	30	7.5
	#7	F-90C	31	30	29	27	25	22	20	18	29	32	8.4

Abbreviations: mel-EDI, equivalent daylight illuminance; EML, equivalent melanopic illuminance; CS, circadian stimulus.

## Data Availability

The data presented in this study are available upon request from the corresponding author.

## Abbreviations

The following abbreviations are used in this manuscript:

VA	Visual acuity
ipRGCs	Intrinsically photosensitive retinal ganglion cells
SPD	Spectral power distribution
mel-EDI	Melanopic equivalent daylight (D65) illuminance
CIE	Commission International de l’Eclairage
EML	Equivalent melanopic lux
CS	Circadian stimulus
CRI	Color Rendering Index
IES	Illuminating Engineering Society
CCT	Correlated color temperature
T	Transmittance T(%)
TCSs	Test Color Samples
Rf	Fidelity Index
CESs	Color evaluation samples
Rg	Gamut Index
SI	International System of Units
AR	Antireflection coating
